# Computational Acoustic Beamforming for Noise Source Identification for Small Wind Turbines

**DOI:** 10.1155/2017/7061391

**Published:** 2017-03-09

**Authors:** Ping Ma, Fue-Sang Lien, Eugene Yee

**Affiliations:** ^1^Department of Mechanical and Mechatronics Engineering, University of Waterloo, Waterloo, ON, Canada N2L 3G1; ^2^Defence R&D Canada, Suffield Research Centre, Stn Main, P.O. Box 4000, Medicine Hat, AB, Canada T1A 8K6

## Abstract

This paper develops a computational acoustic beamforming (CAB) methodology for identification of sources of small wind turbine noise. This methodology is validated using the case of the NACA 0012 airfoil trailing edge noise. For this validation case, the predicted acoustic maps were in excellent conformance with the results of the measurements obtained from the acoustic beamforming experiment. Following this validation study, the CAB methodology was applied to the identification of noise sources generated by a commercial small wind turbine. The simulated acoustic maps revealed that the blade tower interaction and the wind turbine nacelle were the two primary mechanisms for sound generation for this small wind turbine at frequencies between 100 and 630 Hz.

## 1. Introduction

Noise is a critical issue affecting the continued development and use of small wind turbines, owing to the fact that small wind turbines are often installed in proximity of residential (populated) areas. However, similar to other emerging industries, noise issues are of secondary concern for small wind turbine manufacturers. Indeed, rightly or wrongly manufacturers still view the fabrication process and the total wind turbine cost (affordability) as the most important issues that need to be considered for the widespread use of small wind turbines [1]. In addition, the lack of regulation with respect to the noise generated by small wind turbines has curtailed research in this area in both academia and industry. Nevertheless, as more and more people are incentivized to install small wind turbines to supply their electricity needs at home, the noise issues accruing from the operation of small wind turbines will become so critical that rigorous policies will need to be formulated by local and federal agencies to govern the permissible (acceptable) sound levels of noise sources generated by use of small wind turbines in residential areas.

In order to resolve the noise issues associated with the operation of small wind turbines, it is important to determine the locations of the primary sources of sound generation on a wind turbine. To this purpose, it is noted that application of a systematic methodology for noise source identification (NSI) would enable the localization of the sound sources on a wind turbine. This, in turn, would allow engineers to redesign the wind turbine (e.g., blades, hub, and tower) in order to reduce (or minimize) the noise generation. Currently, the NSI methodology for small wind turbines relies mainly on the use of acoustic beamforming measurements. This experimental methodology for noise source determination utilizes arrays of microphones in various geometric configurations for the measurement of the sound field generated by the wind turbine. Subsequently, these array-based microphone sound measurements are processed using high-resolution acoustic beamforming algorithms for the noise source identification. However, the cost of conducting an acoustic beamforming measurement campaign for the noise characterization of a wind turbine is high, especially when it is necessary to use a complex array involving a very large number of microphones. Furthermore, it is frequently difficult to deploy a microphone array at the optimal measurement location for the noise source identification, owing to some environmental limitations (obstacles such as trees, rocks, buildings, etc.). As a consequence, there are very few researchers that have conducted acoustic beamforming measurements for small wind turbines [2–4].

In view of the limitations arising from the use of experimental acoustic beamforming for the noise characterization of small wind turbines, we propose an alternative methodology in this paper. More specifically, we propose to use a computational acoustic beamforming (CAB) methodology for the identification of noise sources on a small wind turbine. The CAB methodology was first proposed by Li [5] where it was used to identify the sources of trailing edge noise for the NACA 0012 airfoil. However, in this application, Li used the methodology to compute only one acoustic map at 800 Hz. This predicted acoustic map was found to be in poor conformance with the corresponding experimental measurement [5]. The poor agreement between the predicted and measured results for the acoustic map was probably due primarily to the coarse mesh used in the computational fluid dynamics (CFD) simulation, which caused an earlier flow separation along the leading edge of the airfoil. This defect, in turn, would have inhibited the interaction between the turbulent boundary layer and the flow in the trailing edge of the airfoil.

To resolve the discrepancies between the experiment and numerical results reported by Li [5] for the NACA 0012 airfoil trailing edge noise, we consider a more computationally demanding and sophisticated implementation of the CAB methodology. Firstly, in our generalization of the CAB methodology, we will use higher fidelity models for the prediction of the flow field. In particular, a large-eddy simulation (LES) with a very fine mesh (*y*^+^ ≤ 1) will be used to compute the flow around the NACA 0012 airfoil in comparison to what was used by Li [5] in his original implementation of the CAB methodology where the flow was determined using an improved delayed detached eddy simulation (IDDES) with a rather coarse mesh (*y*^+^ > 30). Secondly, with reference to certain conclusions reached in some experimental acoustic beamforming studies [6–9], we extend the CAB methodology described by Li [5] to include a diagonal removal process for acoustic beamforming and to include various geometric configurations for the microphone array. Thirdly, we generalize the CAB methodology to incorporate a spherical wave incidence acoustic beamforming algorithm and apply the methodology to cases where the microphone arrays were located in the near and/or transition regions of the sound sources. Fourthly, the CAB methodology is generalized with the inclusion of the most general formulation of the Ffowcs Williams and Hawkings (FW-H) equation for acoustic (sound) propagation: namely, the permeable formulation of the FW-H equation. The generalization of the CAB methodology considered herein will allow the application of this methodology for noise source identification to more complex problems (e.g., localization of noise source generation on a wind turbine and other complicated turbomachinery).

This paper is organized as follows. In Section 2, we describe the framework and models used in the CAB methodology. In Section 3, we validate the methodology using the case of flow over the NACA 0012 airfoil. In Section 4, we apply the CAB methodology to a commercial small wind turbine and identify/analyze the sources of noise generated by this turbine. Finally, in Section 5, we draw some conclusions and discuss some of the advantages of the CAB methodology.

## 2. Computational Acoustic Beamforming Framework

The CAB methodology consists of three components: namely, the CFD, acoustic propagation and acoustic beamforming components. The CFD component is used to simulate the unsteady flow field containing the sound sources; the acoustic propagation component is used to simulate the sound propagation and calculate the acoustic signals at specified locations (e.g., microphone locations); and the acoustic beamforming component is used to generate the acoustic maps using the predicted acoustic (microphone) signals. A hybrid method is used in the CAB methodology for the simulation of the flow-generated noise. This approach decouples the flow simulations (CFD component) from the acoustic calculations (acoustic propagation component). Aerodynamic properties obtained from the CFD simulation can be used as inputs to the acoustic calculations. However, any changes in the noise simulation will not affect the flow field calculations. In this way, the same set of data (sound source information) obtained from the CFD simulation can be used for different arrangements of the receivers. The CFD simulation is the most computationally intensive component of the CAB methodology and, as a result, the use of a hybrid method increases the computational efficiency significantly. This is due to the fact that the CAB methodology can be applied to various cases using different microphone arrangements and/or different microphone array locations without having to redo the CFD calculations. However, the use of this hybrid method in the CAB methodology limits its principal application to flows at low Mach numbers (weakly compressible flows) [10].

**Figure 1:**
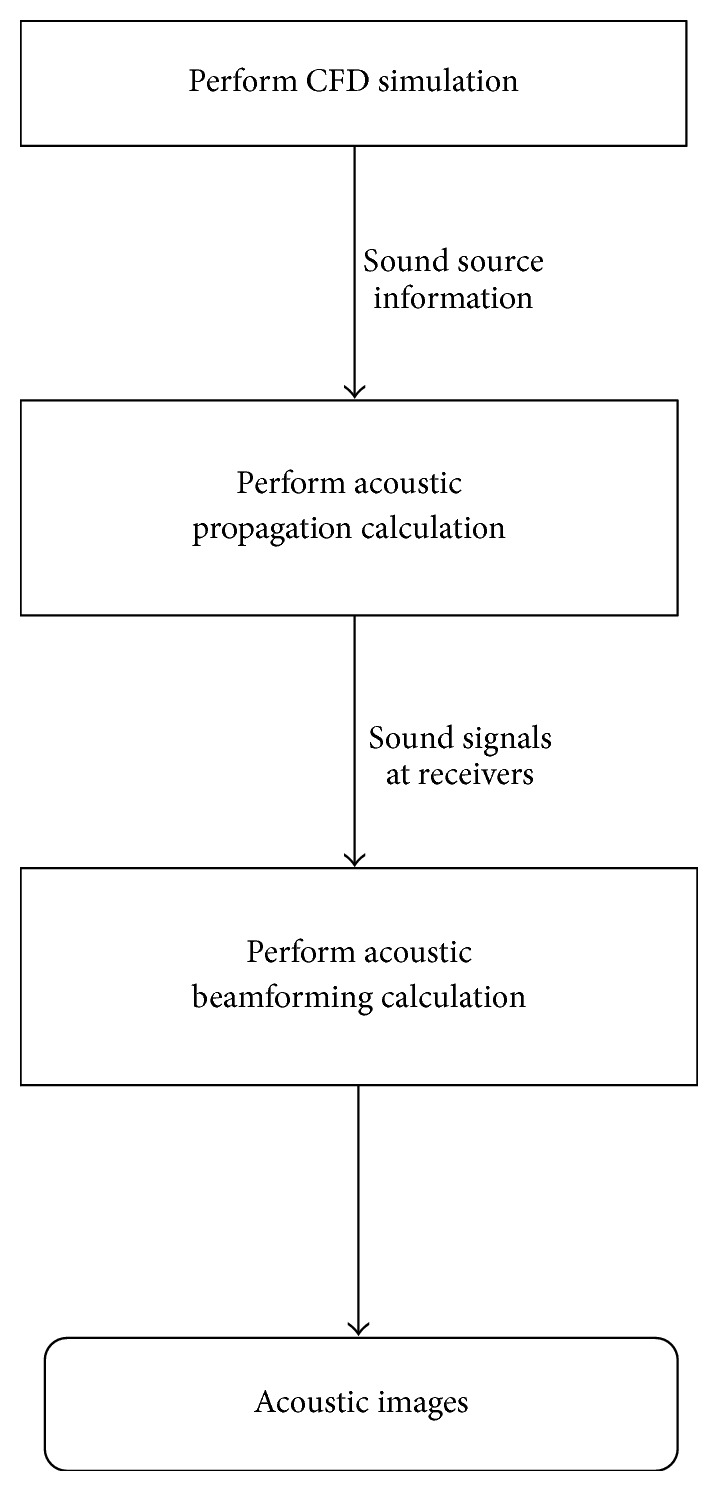
Flowchart showing the three main components of the CAB methodology.

In greater detail, Figure 1 depicts the various components of the CAB methodology. The transient CFD simulation calculates the flow properties (pressure, velocity, density, etc.) at discretized mesh points in the computational domain. The computational domain contains the sound source data acquisition surfaces required for the acoustic propagation calculations. The flow properties obtained on the acoustic acquisition surfaces are utilized by the acoustic propagation solver to calculate the sound signals at the specified microphone (receiver) locations. The acoustic beamforming component utilizes these calculated acoustic signals to generate the acoustic maps at a prescribed source plane. The resulting acoustic maps embody the information on the sound source locations as seen from the microphone array for a specified range of frequencies or for a specified set of octave bands. In this way, the CAB methodology utilizes the virtual microphone signals, which are computationally generated from the information provided by the CFD and acoustic propagation components, for the acoustic beamforming calculation. This is in contrast to conventional acoustic beamforming which employs the microphone signals measured in an actual experiment.

### 2.1. CFD Component

The CFD component involves conducting the CFD simulations to determine the flow field quantities that embody the sound source information, such as the flow velocity, density, and pressure. In the NACA 0012 airfoil and WINPhase 10 wind turbine cases (described later), the flow field around the airfoil or the small wind turbine is simulated in the CFD component.

The CFD simulations are conducted using a commercial CFD package, namely, STAR CCM+®. To this purpose, the flow field around the NACA 0012 airfoil (described in Section 3) was computed using large-eddy simulation [11] with the Smagorinsky subgrid-scale model [12] and the standard Van Driest damping function [13]. Owing to computational resource limitations, the flow field around a small wind turbine (described in Section 4) was computed using the less computationally demanding delayed detached eddy simulation (DDES) methodology [14]. This methodology utilizes the standard Spalart-Allmaras (S-A) turbulence model [15] with rotation/curvature corrections [16] and with a damping function incorporated in the deformation parameter term [17].

### 2.2. Acoustic Propagation Component

The sound source information provided by the CFD component is used by the acoustic propagation component to calculate the sound signals at a set of prescribed locations for the microphone. The acoustic propagation component was conducted using an in-house code that implements the FW-H integral method [18] for computational aeroacoustics. The original FW-H formulation utilized generalized functions to recast the continuity and momentum transport equations into the form of an inhomogeneous wave equation. This formulation includes the effects of very general types of surfaces and motions in the turbulent flow field for the generation of noise. To realize the advantages of the FW-H equation, an integral formulation of the equation can be obtained by convolving the FW-H partial differential equation with the free-space Green's function. The most general form of the FW-H integral formulation is the permeable formulation which has the following form:

(1) $$\begin{array}{c} p^{\prime }\left(\;x,t\;\right)=p_{T}^{\prime }\left(\;x,t\;\right)+p_{L}^{\prime }\left(\;x,t\;\right), \end{array}$$

where

(2) 4πpT′x,t=∫f=0ρ0U˙n+Un˙r1−Mr2retdS+∫f=0ρ0UnrM˙r+cMr−M2r21−Mr3retdS,4πpL′x,t=1c∫f=0L˙rr1−Mr2retdS+∫f=0Lr−LMr21−Mr2retdS+1c∫f=0LrrM˙r+cMr−M2r21−Mr3retdS.

Here,

(3) Lr=Liri,LM=LiMi,Un=Uini,Un˙=Uin˙i,Mr=Miri,

(4) Ui=1−ρρ0vi+ρuiρ0,Mi=vic,Li=p′δijnj+ρuiun−vn,

where *n*_*i*_ is the *i*th component of the unit outward vector normal to the integration surface and *f* = 0 represents the surfaces surrounding the permeable domain of the computational domain. The descriptor “permeable” used in relation to this equation refers to the fact that the surface can be placed outside the solid body, allowing the fluid to flow through it. Furthermore, *r*_*i*_ is the *i*th component of the unit radial vector (**x** − **y**)/*r*, where (**x**, *t*) and (**y**, *τ*) are the receptor and source space-time variables, respectively. The *i*th component of the velocity *v* at the points on the integration surface is denoted by *v*_*i*_, and *u*_*i*_ is the *i*th component of the velocity *u* at points in the local fluid. The *i*th component of Mach number of a point on the boundary surface is denoted by *M*_*i*_ = *v*_*i*_/*c*; *v*_*n*_ is the local normal velocity of the integration surface; and *u*_*n*_ is the local fluid velocity in the direction normal to the boundary surface. The dots over the quantities denote temporal derivatives with respect to the source time *τ*. The subscript ret indicates that the quantity is evaluated at the retarded time *τ* = *t* − *r*/*c*, and the subscripts *T* and *L* denote the thickness and loading noise, respectively.

When the permeable integration surface coincides with the solid surface, the body and fluid velocities are related by *u* = *v* and the impermeable formulation of the FW-H equation is obtained. This formulation is used for the NACA 0012 airfoil simulation. Here, the airfoil surface serves as the sound integration surface and (1) and (2) reduce to the following simpler form:

(5) $$\begin{array}{c} p^{\prime }\left(\;x,t\;\right)=p_{T}^{\prime }\left(\;x,t\;\right)+p_{L}^{\prime }\left(\;x,t\;\right), \end{array}$$

where

(6) 4πpT′x,t=∫f=0ρ0v˙n+vn˙r1−Mr2retdS+∫f=0ρ0vnrM˙r+cMr−M2r21−Mr3retdS,4πpL′x,t=1c∫f=0L˙rr1−Mr2retdS+∫f=0Lr−LMr21−Mr2retdS+1c∫f=0LrrM˙r+cMr−M2r21−Mr3retdS.

### 2.3. Acoustic Beamforming Component

The sound signals calculated with the acoustic propagation component are transferred to the acoustic beamforming component. Within the CAB methodology, the latter component is used to generate the acoustic maps for the identification of the possible sound sources.

The acoustic beamforming calculation is conducted using an in-house code that implements the time-domain delay-and-sum acoustic beamforming algorithm [19]. The spherical wave incidence for the time-domain delay-and-sum acoustic beamforming algorithm assumes the following form:

(7) $$\begin{array}{c} b\left(\;t\;\right)=\overset{MI}{\underset{m=1}{\sum }}w_{m}p_{m}\left(\;t-\Delta_{m}\;\right), \end{array}$$

where the time delay for spherical wave incidence Δ_*m*_ is given by

(8) $$\begin{array}{c} \Delta_{m}=\frac{s-s_{m}}{c}. \end{array}$$

Here, *s* is the distance between the assumed source and the microphone array center and *s*_*m*_ is the distance between the assumed source and the microphone *m*. The spherical wave incidence formulation utilizes the actual wave travel distance to calculate the time delays for each microphone. It is used when the microphone array is located in the near-field and/or transition regions of the sound sources.

The diagonal removal technique, which is widely used to improve the signal-to-noise ratio (SNR) for large microphone arrays mounted on wind-tunnel wall surfaces (e.g., for the removal of turbulent boundary layer wall-pressure fluctuations) [20], has been implemented in our in-house acoustic beamforming code. The spherical wave incidence formulation with diagonal removal [21] is given by

(9) $$\begin{array}{c} b\left(\;\vartheta_{i}\;\right)\text{=}\langle \;{\left[\;\sum_{m=1}^{MI}p_{m}\left(\;{t-\Delta }_{im}\;\right)\;\right]}^{2}+\overset{MI}{\underset{m=1}{\sum }}p_{m}^{2}\left(\;t-\Delta_{im}\;\right)\;\rangle \text{,} \end{array}$$

where *ϑ*_*i*_ represents grid point *i* at the source plane, Δ_*im*_ is the propagation time from the source plane grid point *i* to the microphone *m*, and 〈  〉 indicates a time averaging operation.

## 3. Validation Using the NACA 0012 Airfoil

The experimental aeroacoustic data for the NACA 0012 airfoil provided by the National Renewable Energy Laboratory (NREL) was used for the validation of the CAB methodology. The objective of this experiment was to understand the aeroacoustic performance of six different airfoils that are candidates for use in small wind turbines [22]. Five acoustic maps for the frequency range from 2000 to 5000 Hz were obtained in the experimental measurements for the NACA 0012 airfoil. This information was used for the identification of the sound source location on the airfoils. The details of the experimental procedure are described in [22]. Because no aerodynamic experimental data were available for the NACA 0012 airfoil measurements reported in [22], two other experiments [23, 24] were selected to provide data that can be used to compare the observed pressure, lift, and drag coefficients with the numerical predictions. The aerodynamic data from these two experiments were conducted at an inlet Mach number Ma = 0.15 (in comparison with an inlet Mach number of Ma = 0.12 for our simulation) and at Reynolds numbers in the range 1.44 × 10^6^ ≤ Re ≤ 9 × 10^6^ (in comparison with a Reynolds number of Re = 0.62 × 10^6^ for our simulation).

### 3.1. Details of the Simulation

#### 3.1.1. Flow Field

The computational domain used for the aerodynamic simulation of the NACA 0012 airfoil has dimensions of 10*C* in the streamwise direction, 6*C* in the wall normal direction, and 0.14*C* in the spanwise direction, where *C* is the airfoil chord length.

**Figure 2:**
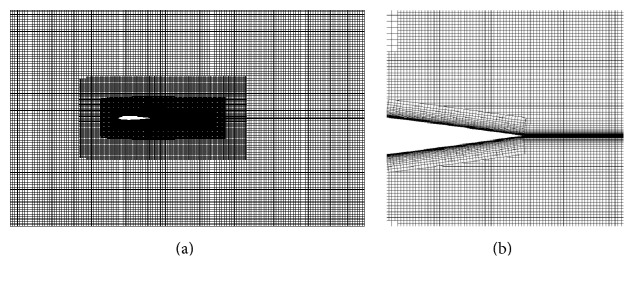
Computational mesh used for the aerodynamic simulation of the NACA 0012 airfoil: (a) mesh for the whole computational domain and (b) mesh around the airfoil trailing edge.

Twenty prism layers were generated around the airfoil with a layer stretch ratio of 1.2. The resulting nondimensional wall distance had the value of *y*^+^ ≤ 1 (recall *y*^+^ ≡ *u*_*τ*_*y*/*ν* where *u*_*τ*_ is the friction velocity and *ν* is the kinematic viscosity of the fluid). A dense mesh was created surrounding the airfoil as shown in Figure 2. An increased spatial resolution was applied around the airfoil trailing edge and wake regions as shown in Figure 2(b). A total of 3.6 million grid nodes were generated in the computational domain.

The aerodynamic simulation of the flow around the NACA 0012 airfoil was based on LES with the Smagorinsky subgrid-scale model [12] and the standard Van Driest damping function [13]. The three-dimensional unsteady spatially averaged Navier-Stokes equations were solved using a cell-centered finite volume method. The gradient approximation of all quantities used in the discretization of the governing equations used a hybrid Gauss-LSQ (least squares) cell-based scheme with the Venkatakrishnan limiter. A bounded central-differencing scheme was employed for discretization of the convective term in the momentum transport equation. A pressure-weighted interpolation scheme was used to estimate the pressure values at the cell faces from their values at the cell centroids. A collocated variable arrangement was used and the Semi-Implicit Method for Pressure-Linked Equations (SIMPLE) method was employed for the pressure-velocity coupling. A second-order implicit scheme was chosen for the time marching algorithm with a fixed nondimensional time step of *t*^*∗*^ = 0.0017, where the nondimensional time step is defined as *t*^*∗*^ ≡ *tU*_ref_/*C*, where *U*_ref_ = 40 m s^−1^ is the (reference) velocity at the computational domain inlet. With this choice of time step, the average Courant-Friedrichs-Lewy (CFL) number was kept below a value of 1 within the entire computational domain. In the solution of the discretized equations, a maximum of 50 iterations was permitted for each time step.

A uniform velocity distribution was prescribed at the inlet of the computational domain with an inlet Mach number of 0.12. The pressure at the outlet boundaries of the domain was set to atmospheric pressure. The airfoil surfaces were treated as no-slip smooth walls. The top and bottom boundaries of the computational domain were treated as symmetric planes. Periodic boundary conditions were applied at the front and back surfaces of the computational domain.

#### 3.1.2. Acoustic Field

The impermeable integration surface used for the aeroacoustic simulation coincides with the airfoil wall boundary. The acoustic propagation calculation used the sound source data which was obtained by periodically extending the original sound source data derived from the CFD simulation by five times in the spanwise direction. The impermeable formulation of the FW-H equation is employed for the aeroacoustic simulation. The central-differencing scheme was used to approximate the time derivatives in the acoustic propagation calculations.

**Figure 3:**
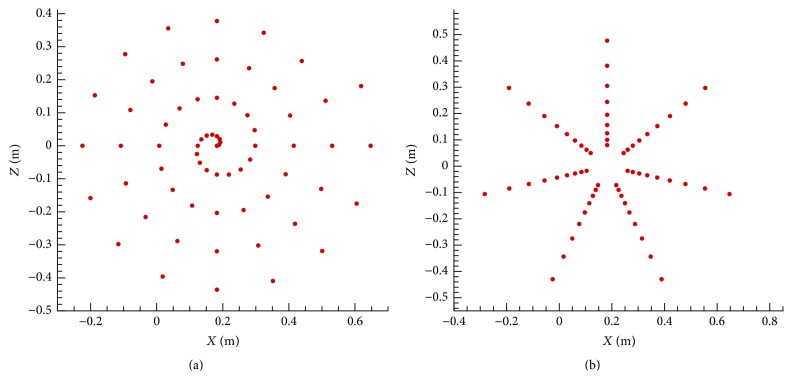
Two microphone arrays used for aeroacoustic simulation for the NACA 0012 airfoil: an Archimedean spiral array (a) and a star array (b).

**Figure 4:**
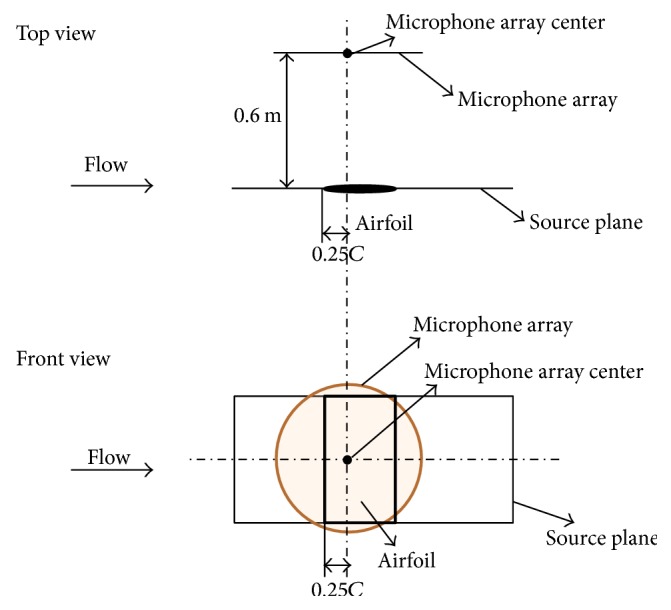
Sketch showing the location of the microphone array and the source plane used for the aeroacoustic simulation of the NACA 0012 airfoil.

The spherical wave incidence acoustic beamformer was used for the acoustic beamforming calculations. Owing to the fact that the microphone array geometry employed in the NACA 0012 airfoil experiment [22] was not described in detail, two different arrays were used in our simulations. The geometry for these two arrays was as follows: (1) an Archimedean spiral array consisting of 66 microphones and (2) a star array consisting of 63 microphones (both of which are shown in Figure 3). The microphone arrays used in our simulation have dimensions of 0.8 m × 0.8 m and were similar in size to the array used in the wind-tunnel experiment which had a dimension of 0.8 m × 0.6 m [22]. These two microphone arrays, which were placed at the same location as in the experiment [22], are centered at a distance of *C*/4 along the airfoil chord and at a distance of around 0.6 m away from the source plane as shown in Figure 4. The array sampling frequency was 100 kHz and the data recording period was 0.5 s during the simulation.

The acoustic images were computed at the source plane with 3 mm spatial resolution in the spanwise direction and 7 mm spatial resolution in the streamwise direction, in conformance with those measured in the wind-tunnel experiment [22]. As in the case of the acoustic images measured in this experiment, the acoustic maps obtained using the CAB methodology were frequency averaged over one-third octave bands. The determination of the center frequencies and the upper and lower frequency boundaries (the band width) that define each one-third octave band can be found in [25]. An arithmetical average is used to calculate the averaged sound pressure level within each one-third octave band. The dimensions of the acoustic images are 3*C* × 0.7*C*.

### 3.2. Results and Analysis

#### 3.2.1. Flow Field

The predicted lift (*c*_*L*_) and drag (*c*_*D*_) coefficients for the NACA 0012 airfoil at zero degree angle-of-attack are compared with two observed results obtained in two experiments [23, 24] as summarized in Table 1. A perusal of Table 1 indicates that there is good agreement between the predicted time-averaged lift and drag coefficients and the corresponding measured quantities obtained from the two experiments.

**Figure 5:**
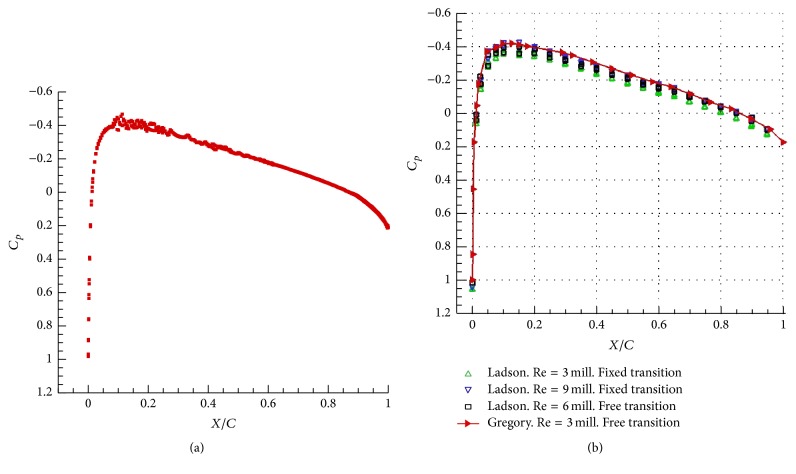
Mean airfoil surface pressure coefficient at zero degree angle-of-attack: predicted results (a) and experimental data (b). The upper triangle symbols correspond to the experimental data from [24] at Re = 3 × 10^6^ with the transition fixed at the 5-percent-chord model station. The down triangle symbols correspond to the experimental data from [24] at Re = 9 × 10^6^ with the transition fixed at the 5-percent-chord model station. The square symbols correspond to the experimental data from [24] at Re = 6 × 10^6^ for a free transition. The right triangle symbols (with the solid line) represent the experimental data from [23] at Re = 3 × 10^6^ for a free transition.

**Figure 6:**
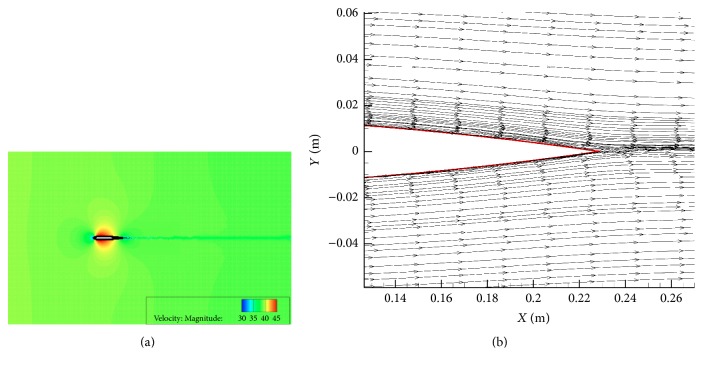
Predicted velocity magnitude contours and streamlines around the NACA 0012 airfoil: (a) velocity magnitude (m s^−1^) contours and (b) velocity streamlines around the trailing edge region of the airfoil.

Figure 5 compares the predicted mean surface pressure with the experimental data [23, 24]. Overall, the numerical prediction agrees very well with the experimental data, except perhaps around region associated with the leading edge of the airfoil. The contours of the velocity magnitude, shown in Figure 6(a), exhibit a symmetric distribution on the upper and lower surfaces of the airfoil. This result is in good agreement with other numerical results reported in the literature [26]. The streamlines around the trailing edge region of the airfoil are displayed in Figure 6(b). A careful perusal of this figure indicates that small-scale turbulence is generated in the boundary layer along the airfoil surface and this turbulence is transported towards the trailing edge of the airfoil where it interacts with the flow in this region.

#### 3.2.2. Aeroacoustic Field

**Figure 7:**
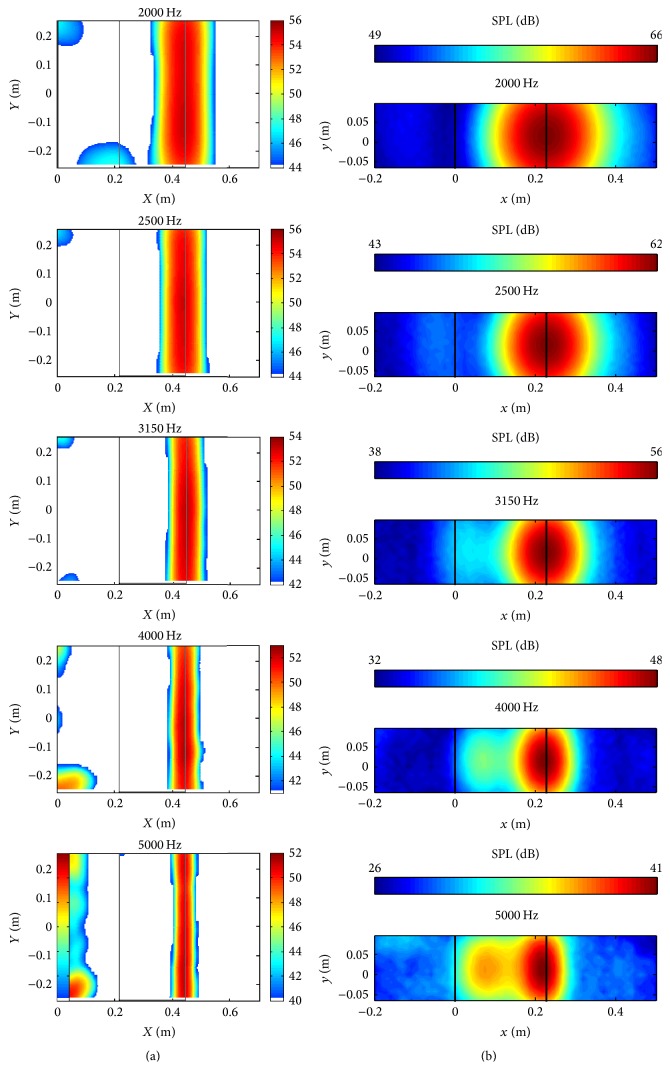
Acoustic maps for the NACA 0012 airfoil obtained from experimental measurements [22] (a) and from the numerical simulation (b).

Figure 7 compares the predicted acoustic maps (b) with the measured acoustic maps of the sound pressure level (SPL) obtained from an experiment described in [22] (a) over the frequency range from 2000 to 5000 Hz (inclusive). These acoustic maps were obtained for the NACA 0012 airfoil. The Archimedean spiral array was employed for this simulation. The diagonal removal process was not used in the acoustic beamforming calculation.

Despite the differences in the sizes and coordinates (different locations were chosen for the origins in the simulation and experiment, resp.) of the acoustic maps shown in Figures 7(a) and 7(b), the maps generated from the numerical simulation are in very good conformance with those generated from the experimental data. The CAB methodology predicts that the sound source is located right at the trailing edge of the airfoil for all the frequencies shown, which agrees well with the experimental measurements. Furthermore, the area of the region of maximum SPL in the acoustic maps decreases as the frequency increases, implying that a better spatial resolution of the source is obtained at the higher frequencies. The same phenomenon is also seen in the experimental results shown in Figure 7(a). This trend can be explained by consideration of the definition of the acoustic beamforming resolution [19]:

(10) $$\begin{array}{c} R_{BF}\approx 1.22\frac{L}{\Pi }\lambda , \end{array}$$

where *R*_BF_ is the spatial resolution, *L* is the measurement distance, Π is the microphone array diameter, and *λ* is the wavelength of interest. From the relationship of (10), it is seen that the spatial resolution *R*_BF_ is proportional to wavelength *λ* (or, equivalently, the spatial resolution is inversely proportional to the frequency *f* since *λ* ≡ *c*/*f*).

It is noted that the acoustic maps obtained from the numerical simulation provide a much larger dynamic range than those obtained from the experimental measurements. This implies that the acoustic maps obtained from the numerical simulations using the CAB methodology have a higher signal-to-noise ratio (SNR) than those obtained from the experimental measurements. This is not surprising given the fact that the experimental data are subject to various sources of noise (e.g., background noise) that is absent in the numerical data.

**Figure 8:**
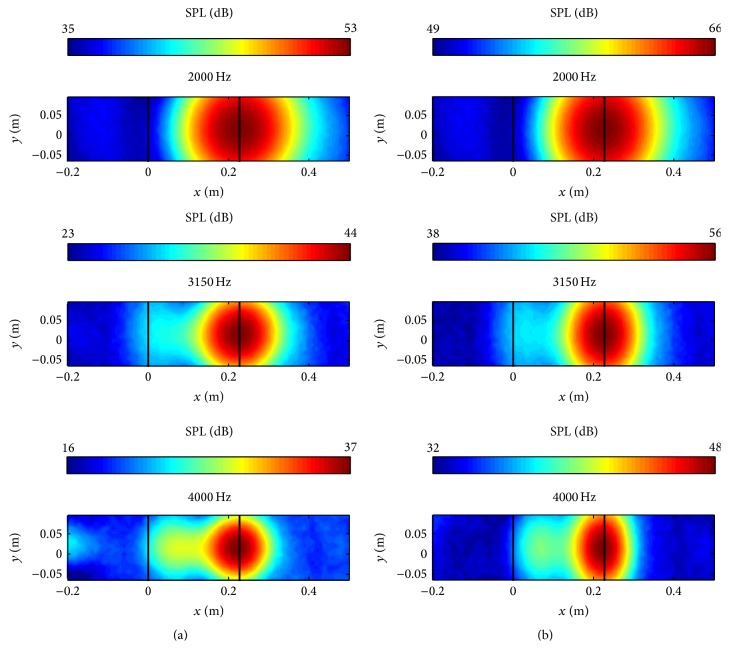
Predicted acoustic maps for the NACA 0012 airfoil obtained using the original sound source data (a) and using a periodic extension of the original sound source data in the spanwise direction (b).

In order to evaluate the effect of the periodic expansion of the sound source information in the spanwise direction on the resulting acoustic maps, Figure 8 compares the acoustic maps obtained using the original sound source data derived from the CFD simulations (a) with those obtained by periodically extending the original sound source data five times in the spanwise direction (b). It is seen that both results predict the location of the trailing edge noise correctly compared with the experimental data as shown in Figure 7(a). In addition, the acoustic maps obtained from the original sound source data and from a periodic extension of this data are seen to have similar dynamic ranges. Nevertheless, it is seen that the acoustic maps generated in Figure 8 for the periodic extension of the sound source (b) have larger sound pressure levels than those for the original sound source (a). Finally, an examination of Figure 8 shows that the detected noise source on the acoustic maps for the periodically extended sound source is elongated along the trailing edge of the airfoil, whereas the detected noise source on the acoustic maps for the original sound source appears to be circular in shape. In summary then, the acoustic maps provided by the periodically extended sound source are generally in better conformance with the experimental measurements than those for the original sound source.

**Figure 9:**
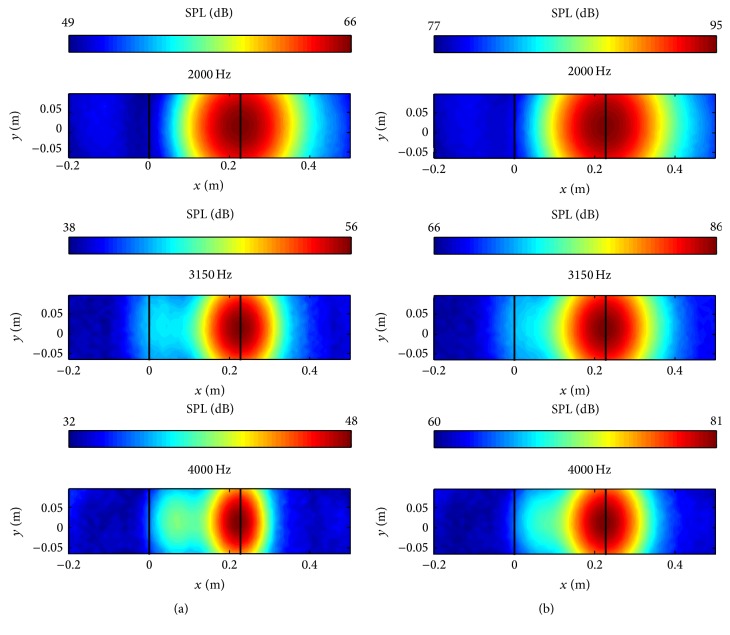
Predicted acoustic maps for the NACA 0012 airfoil obtained without (a) and with (b) the inclusion of the diagonal removal process in the acoustic beamforming calculations.

Continuing with the validation of the CAB methodology using the NACA 0012 airfoil, we study the effect of the inclusion of the diagonal removal process in the acoustic beamforming calculations on the generation of the acoustic maps.  Figure 9(a) shows the acoustic maps obtained without the inclusion of the diagonal removal process in the acoustic beamforming calculations, whereas Figure 9(b) shows the acoustic maps obtained with the inclusion of the diagonal removal process. The sound source locations on the acoustic maps obtained both with and without the inclusion of the diagonal removal technique in the acoustic beamforming are very similar. A perusal of Figure 9(b) shows that the use of the diagonal removal technique increases the dynamic range of acoustic maps as the frequency increases. A similar result regarding the increase of the dynamic range on acoustic maps obtained with the inclusion of the diagonal removal technique as the frequency increases has also been reported in the literature [9].

Furthermore, the areas of the region of maximum SPL on the acoustic maps with the inclusion of the diagonal removal technique are slightly larger at the higher frequencies than those obtained without the inclusion of the diagonal removal technique. This shows that the diagonal removal process might worsen the acoustic beamforming spatial resolution while increasing the dynamic range. Although most of the investigations reported that the use of the diagonal removal technique improved the dynamic range on the acoustic maps, some investigators [6, 8, 9] suggest that caution needs to be taken in the inclusion of the diagonal removal technique in acoustic beamforming, especially for cases involving the identification of multiple sources. Since the NACA 0012 airfoil results without the inclusion of the diagonal removal agreed very well with the experimental data and the diagonal removal technique is itself a computational demanding process in the acoustic beamforming component, the acoustic images reported henceforth in this paper will be generated without the inclusion of the diagonal removal technique in the acoustic beamforming calculations.

**Figure 10:**
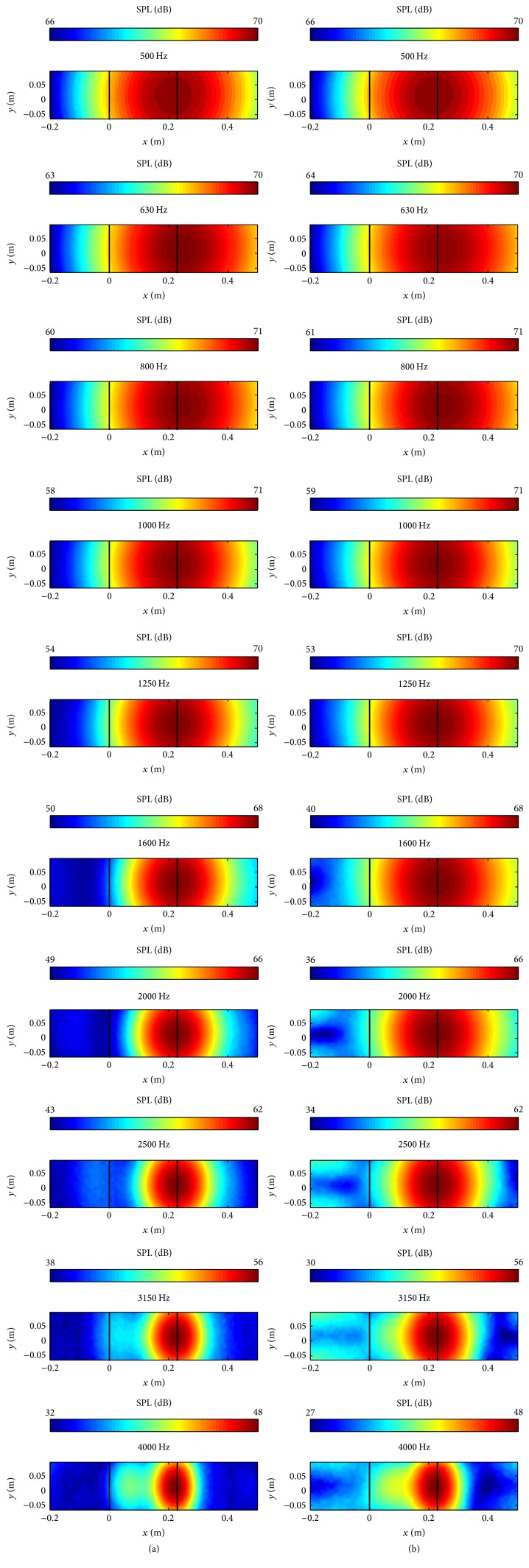
Predicted acoustic maps for the NACA 0012 airfoil obtained using an Archimedean spiral array (a) and a star array (b).

Next, we investigate the effect of different microphone array geometries on the generation of acoustic maps.  Figure 10(a) displays the acoustic maps obtained for an Archimedean spiral array and (b) shows those obtained for a star array for a frequency range extending from 500 to 4000 Hz, inclusive. Firstly, the acoustic maps for both microphone arrays identified the trailing edge of the airfoil as the location of the sound source, which agrees well with the experimental measurements (cf. Figure 7(a)). Consequently, both microphone array geometries yielded a correct identification of the noise source for the NACA 0012 airfoil. Secondly, at all the frequencies investigated, the sound source area predicted using an Archimedean spiral array is smaller than that predicted using a star array. This indicates that the Archimedean spiral array has better spatial resolution than the star array on the acoustic maps for the NACA 0012 airfoil. Thirdly, the dynamic ranges of the acoustic maps obtained for the Archimedean spiral array are slightly larger than those obtained for the star array for frequencies below 1000 Hz. At frequencies above 1000 Hz, a star array produces a much wider dynamic range in the acoustic maps than an Archimedean spiral array. This implies that an Archimedean spiral array results in a higher SNR for frequencies below 1000 Hz, but a lower SNR for frequencies above 1000 Hz, than a star array. Similar conclusions were reached in [7] where an aeroacoustic test of a larger NACA 0012 airfoil (longer span and chord length) was conducted experimentally.

It is stressed that there is no “universal” microphone array geometry that would produce optimal results vis-à-vis the acoustic beamforming for every case. For the current application involving the identification of trailing edge noise from the NACA 0012 airfoil, an Archimedean spiral array generally resulted in a better spatial resolution, but a star array yielded a higher SNR at frequencies above 1000 Hz. However, it is not possible to conclude that one array performs better than the other because both arrays were found to provide correct predictions of the locations of the sound source for the various frequencies examined. In consequence, both array geometries will be used to generate acoustic maps for the identification of the source of wind turbine noise described in the next section.

## 4. Application to the WINPhase 10 Wind Turbine

The flow field simulation and acoustic propagation calculations for the WINPhase 10 small wind turbine have been conducted previously by Ma et al. [27]. In this study, both the wind turbine power output and the A-weighted SPL spectra were compared with some field measurements of these quantities for this wind turbine [28]. It was found in [27] that the numerical predictions of the power and of the SPL spectra were generally in very good conformance with the available field measurements. In the context of the current paper, the numerical results for the aerodynamic and aeroacoustic predictions undertaken in [27] for the WINPhase 10 wind turbine will be used in the third stage (acoustic beamforming component) of the CAB methodology for the generation of the acoustic maps required for the noise source identification. The results of this analysis will be reported in this section. However, for completeness, we will first provide a brief description of the CFD and acoustic propagation calculations [27], as well as the measurements of the sound pressure levels [28], that were undertaken for the WINPhase 10 wind turbine.

The WINPhase 10 wind turbine is a three-bladed upwind-arranged small wind turbine. The field measurements of the sound pressure level for this wind turbine were conducted by Intertek Testing Services Ltd. [28] at the wind farm Sunite located in the northern part of China. These measurements were made over a period of two days. The measured A-weighted SPL was averaged and separated from the background noise for each one-third octave band for wind speeds in the range from 4 to 11 m s^−1^. The resulting A-weighted SPL for each one-third octave band at each wind speed and the details of the measurement equipment are summarized in a field measurement report [28]. The A-weighting method associated with the measurement of the sound pressure level (which accounts for the relative loudness as perceived by the human ear) is described in [25].

### 4.1. Details of the Simulation

#### 4.1.1. Flow Field

**Figure 11:**
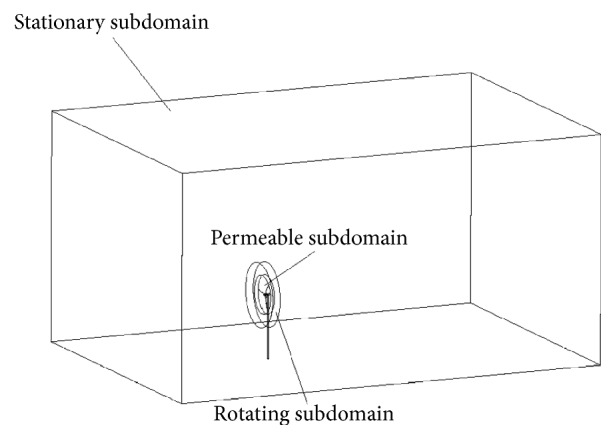
Computational domain used for the aerodynamic simulation of the WINPhase 10 small wind turbine.

Figure 11 depicts the computational domain used for the aerodynamic simulation of the WINPhase 10 wind turbine. The computational domain is partitioned into three subdomains. These subdomains include a permeable subdomain, a rotating subdomain, and a stationary subdomain. The permeable subdomain includes the rotor and was utilized for the sound source data acquisition (which is the input required for the subsequent aeroacoustic calculations).

**Figure 12:**
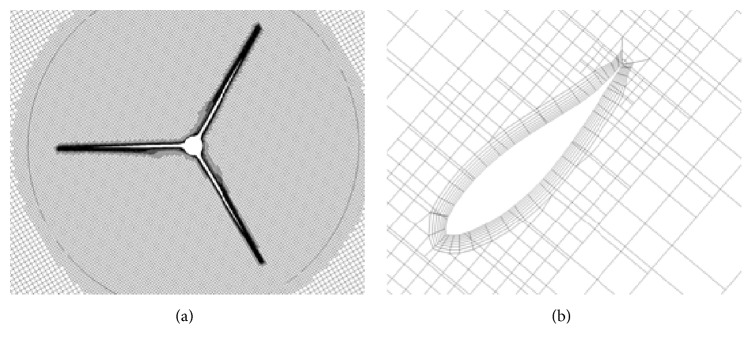
The mesh used for the discretization of the computational domain for the WINPhase 10 wind turbine aerodynamic simulation: (a) front view of the three blades and (b) seven prism layers surrounding a turbine blade.

The dimensions of the computational domain were as follows: 14*D* in the streamwise direction, 9.6*D* in the spanwise direction, and 7*D* in the vertical direction. Here, *D* is the WINPhase 10 wind turbine rotor diameter. The entire computational domain was discretized using about 4.34 million cells, with about 1.6 million of these cells concentrated in the permeable subdomain as illustrated in Figure 12. Moreover, seven prism layers were applied around each blade in order to resolve the flow here.

As mentioned earlier, the CFD simulation for the WINPhase 10 wind turbine applied the DDES methodology using the S-A turbulence model [15] with rotation/curvature corrections [16] and with the inclusion of a damping function correction in deformation parameter [17]. A first-order upwind scheme was used to approximate the convective term in the transport equation for the turbulence viscosity (namely, in the S-A turbulence model). The diffusion terms in all the other transport equations were discretized using a central-differencing scheme. A second-order implicit scheme was used for the time advancement. A fixed time step of 0.0001 s was used for the time advancement. For this choice of time step, the average CFL number was less than one over the whole computational domain.

A set of reference wind speeds in the range from 9 to 11 m s^−1^ with a one-seventh power-law dependence on height above the ground surface was used to prescribe the wind speed profile at the computational domain inlet for the DDES simulations. The turbulence viscosity ratio was set to a value of 10 at the inlet boundary [29]. The pressure at the domain outlet boundary was set to the atmospheric pressure. The surfaces of the wind turbine rotor and tower were treated as no-slip smooth walls.

#### 4.1.2. Acoustic Field

**Figure 13:**
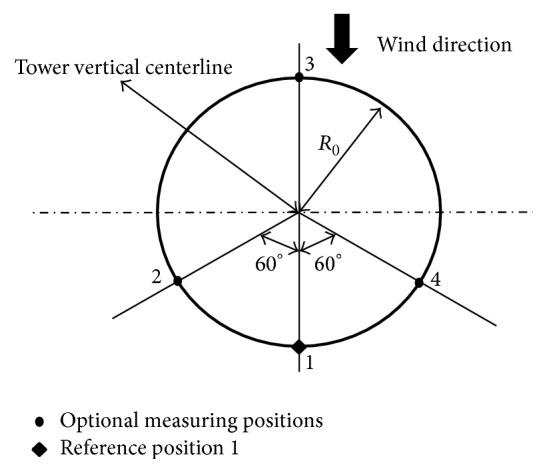
Standard configuration for microphone measurement positions (plan view).

In accordance with the American Wind Energy Association (AWEA) and the International Electrotechnical Commission (IEC) standards [30, 31], for the measurement of sound pressure levels emitted from a wind turbine, a microphone needs to be placed at the reference location shown in Figure 13. Both the field measurements [28] and numerical simulations using the CAB methodology place the microphone at the reference position indicated in Figure 13.

The acoustic propagation solver (second component of the CAB methodology) used the data (sound source information) obtained from the aerodynamic simulation of three complete revolutions of the WINPhase 10 wind turbine blades. The permeable formulation of the FW-H equation was used for the sound propagation calculation. The Stirling scheme [32] was employed for the discretization of the time tendency term in the permeable FW-H equation. A-weighting was applied to the predicted acoustic spectra followed by frequency averaging over one-third octave bands.

**Figure 14:**
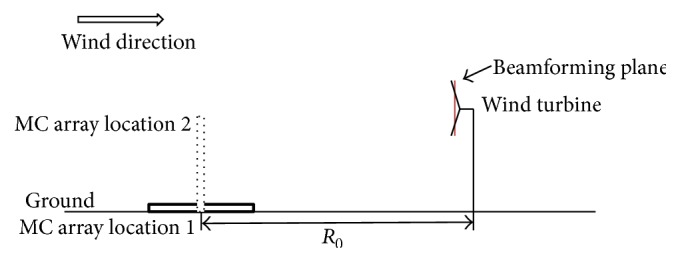
Sketch showing the locations of the microphone (MC) arrays used for the acoustic beamforming for the WINPhase 10 wind turbine.

Figure 14 shows a sketch of the numerical acoustic beamforming setup for the WINPhase 10 wind turbine. The center of the microphone array was placed upstream of the wind turbine at a distance of *R*_0_ where *R*_0_ = *H* + *D*/2 (*H* is the hub height) [30, 31]. The acoustic beamforming source plane coincides with the wind turbine rotor plane and has a size of 6 m × 6 m. The grid used for the acoustic beamforming has 121 points in both directions resulting in the generation of acoustic maps with 14,641 points. A spherical wave incidence acoustic beamformer was employed without the use of the diagonal removal process. The sampling frequency is 10 kHz and the sampling duration corresponds to three complete revolutions of the wind turbine blades. No weighting method is applied to the microphone signals used in the acoustic beamforming calculation.

Two microphone array geometries (namely, an Archimedean spiral array and a star array) were used for the acoustic beamforming calculations for the WINPhase 10 wind turbine. Three scenarios, which were determined by the lowest frequency of interest and by the focusing capabilities of the array, were applied for each array geometry: (1) an 8 m × 8 m horizontal array placed on the ground; (2) a 20 m × 20 m horizontal array placed on the ground; and (3) a 20 m × 20 m vertical array oriented parallel with the wind turbine rotor plane.

### 4.2. Results and Analysis

#### 4.2.1. Aerodynamic Results and Analysis

**Figure 15:**
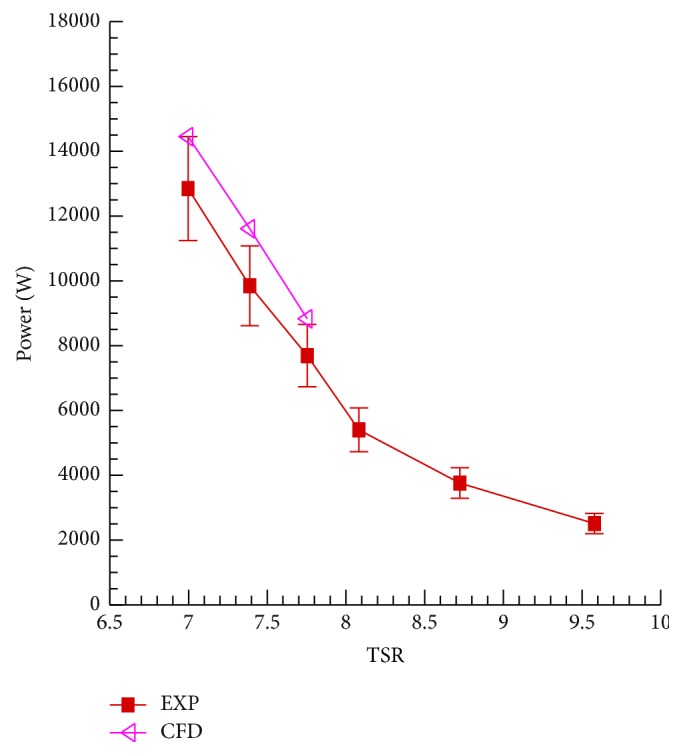
WINPhase 10 wind turbine power predictions compared with field measurement data.

Figure 15 compares the power curves for the WINPhase 10 wind turbine obtained from our numerical simulations with the corresponding experimental data for a range of values of the tip speed ratio (TSR). The field measurements of the wind turbine power output were conducted by the wind turbine manufacturer WINPhase Energy Inc. and the power curve measurement data were obtained from the manufacturer (pers. comm.). The experimental data used here were obtained from full-scale wind turbine field measurements, rather than from a well-controlled wind-tunnel study. The error bars shown in Figure 15 only reflect the uncertainty of the generator efficiency. Other sources of uncertainty arising from the inflow turbulence and the effects of the complex terrain were very difficult to quantify. Owing to the fact that the numerical simulations were performed in an idealized environment with none of the complications that were present in the actual field measurements, it is expected that the turbine power predictions from our simulations should be larger than the actual turbine power measured in the field experiments where numerous environmental factors would have resulted in power losses. Hence, in view of this consideration, the over-prediction of the power by the numerical simulations in Figure 15 is not surprising.

#### 4.2.2. Aeroacoustic Results and Analysis

**Figure 16:**
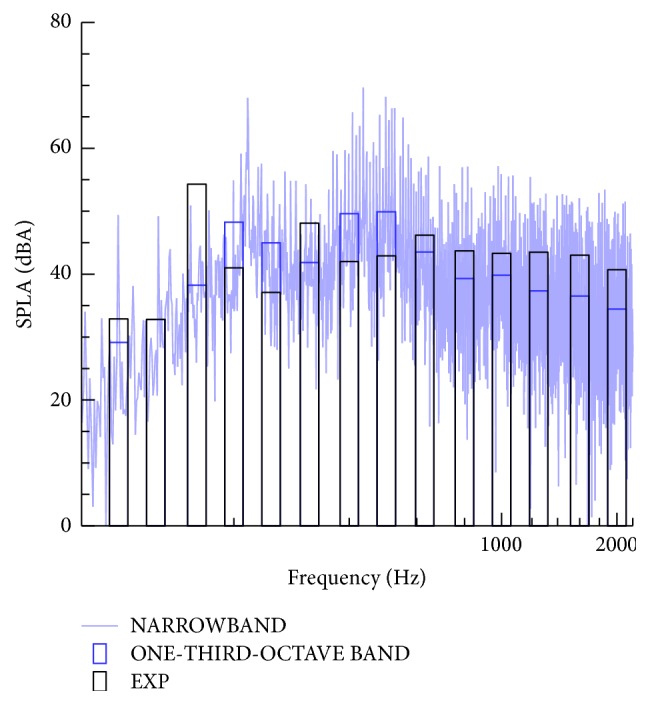
Spectra of the A-weighted sound pressure level (SPLA) for an inflow velocity of 9 m s^−1^ at hub height. The continuous lines show the narrow-band SPLA spectra. The bars correspond to the SPLA spectra frequency averaged over one-third octave bands (blue bar: numerical results; black bar: experimental data (EXP)).

Figure 16 compares the magnitude of the predicted A-weighted sound pressure level (SPLA) spectra averaged over one-third octave bands with the associated experimental measurements [28] at the reference location for an inflow wind speed of 9 m s^−1^ at the hub height for the WINPhase 10 wind turbine. In addition, this figure displays the corresponding narrow-band (namely, with no frequency averaging) SPLA spectra from our aeroacoustic simulations. It is seen that the numerical predictions for the magnitude of the SPLA frequency averaged over one-third octave bands (blue horizontal bars) agree well with the corresponding experimental measurements (black horizontal bars). Nevertheless, the wind turbine noise level above 1000 Hz appears to be underpredicted. This may be the result of the numerical dissipation inherent in the discretization of the convective terms in the momentum transport equation (namely, in the CFD simulations that provide the input data needed for the subsequent acoustic predictions). Similar findings were obtained in the cases for inflow velocities of 10 m s^−1^ and 11 m s^−1^.

**Figure 17:**
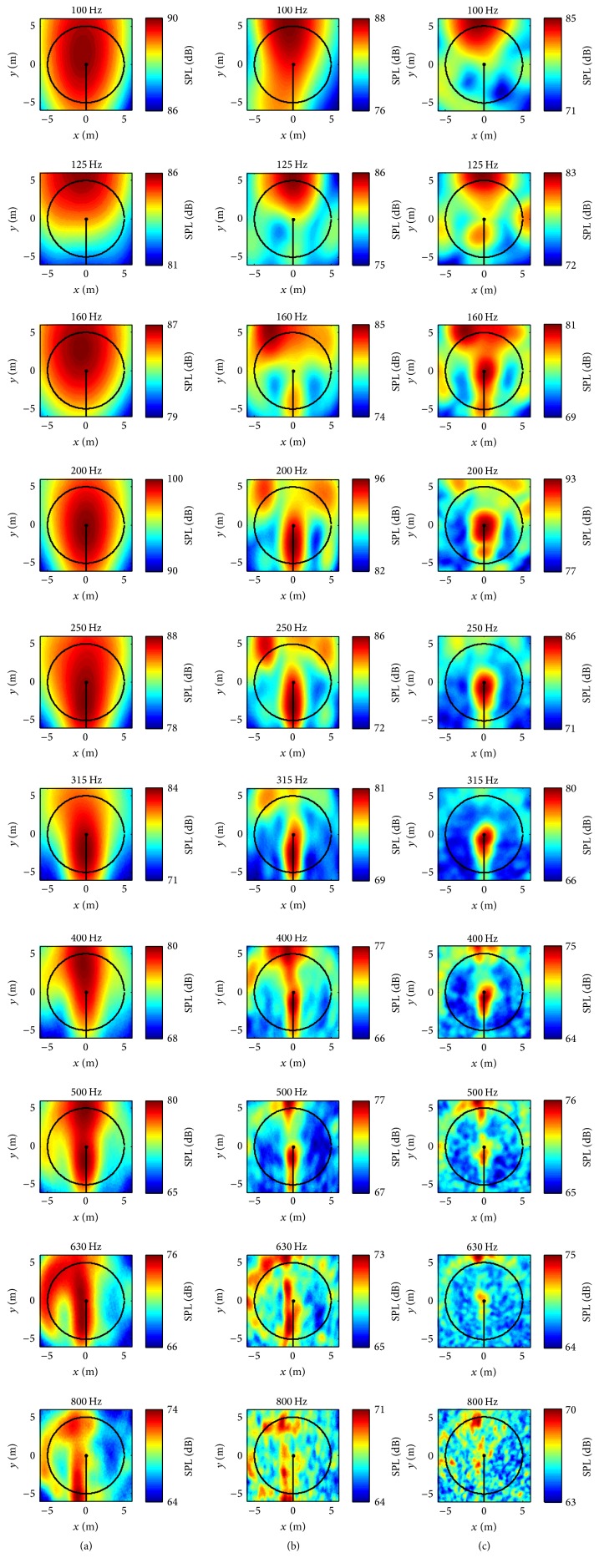
Predicted acoustic maps for the WINPhase 10 wind turbine for an Archimedean spiral array: 8 m × 8 m horizontal array at ground level (a); 20 m × 20 m horizontal array at ground level (b); 20 m × 20 m vertical array parallel to the wind turbine rotor plane (c). The inflow velocity is 9 m s^−1^ at the turbine hub height. The wind turbine rotates in the counter-clockwise direction.

**Figure 18:**
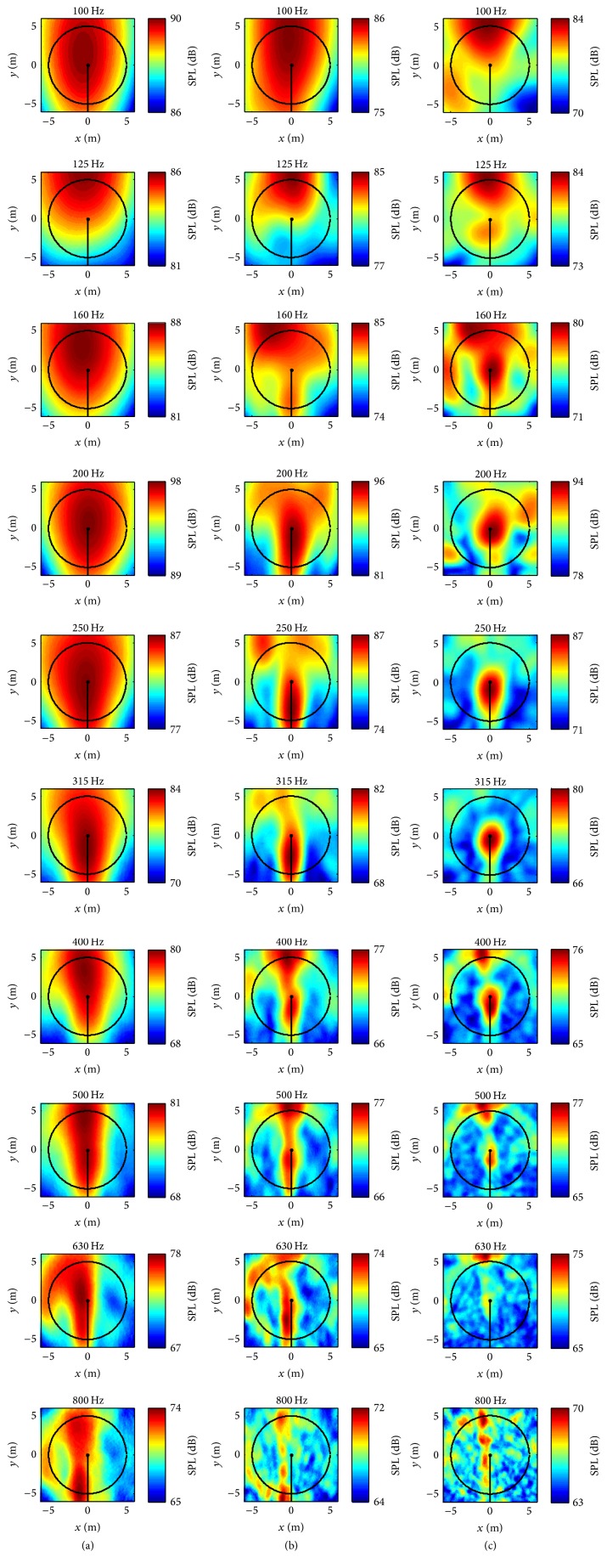
Predicted acoustic maps for the WINPhase 10 wind turbine for a star array: 8 m × 8 m horizontal array at ground level (a); 20 m × 20 m horizontal array at ground level (b); 20 m × 20 m vertical array parallel to the wind turbine rotor plane (c). The inflow velocity is 9 m s^−1^ at the turbine hub height. The wind turbine rotates in the counter-clockwise direction.

The acoustic maps for three inflow wind speeds (9 m s^−1^, 10 m s^−1^, and 11 m s^−1^ at the wind turbine hub height) were generated from the predicted sound signals at the microphones for two array geometries. Owing to similarities of the acoustic maps obtained for the three inflow velocities, we present only the acoustic maps generated for a 9 m s^−1^ inflow velocity. These maps were computed in the frequency range from 100 to 800 Hz for one-third octave bands (the same bands as displayed in Figure 16 for the SPLA frequency averaged spectra). The results for an Archimedean spiral array and for a star array are exhibited in Figures 17 and 18, respectively.

According to (10), at a fixed measurement distance, the spatial resolution of the acoustic map is proportional to the wavelength but inversely proportional to the array size. This dependence is evident from a careful perusal of the acoustic maps exhibited in Figures 17 and 18. For both the Archimedean spiral and the star arrays, the area of the identified sound source in the acoustic maps is seen to decrease as the frequency increases. This implies that the localization of the source is better resolved spatially as the frequency increases. Furthermore, the sound source is better localized spatially using the 20 m × 20 m array in comparison to that obtained using the 8 m × 8 m array (cf. Figures 17(a), 17(b), 18(a) and 18(b)). As a consequence, it is seen that the spatial resolution of a putative sound source increases as the size of the microphone array increases. Similar conclusions were also reported in the literature [33] for experimental measurements.

For the NACA 0012 airfoil case investigated in Section 3, both the numerical results and the literature [7] suggest that an Archimedean spiral array provides a better spatial resolution of a sound source in the acoustic map than that obtained from a star array. This conclusion also holds for the current case involving a small wind turbine. More specifically, this is evident on comparing the acoustic maps in Figure 17 (Archimedean spiral array) with those in Figure 18 (star array). Nevertheless, the sound sources identified using both the Archimedean spiral and star arrays occur at similar locations in the acoustic maps.

For the two different microphone array geometries employed, the area of the identified sound source in the acoustic maps obtained for the vertical microphone array (Figures 17(c) and 18(c)) is smaller than that identified by the same size horizontal microphone array at ground level (Figures 17(a) and 18(a)), implying that a better resolution was achieved with the vertical microphone array. This is because the opening angle of the acoustic beamformer is decreased from 50° to 22° by using the vertical microphone. According to the literature [19], a useful opening angle for an acoustic beamformer in practice is restricted to 30°. Furthermore, the smaller the opening angle, the better the spatial resolution that is achieved in the resulting acoustic maps. However, for actual acoustic beamforming measurements, erecting a large-sized microphone array is usually very difficult (technically and logistically). This is the reason that most of the large-sized microphone arrays are placed on the ground during measurements, with the disadvantage that this leads to a large opening angle for the acoustic beamformer. On the other hand, these limitations for the installation of microphone arrays are absent when the CAB methodology is used for the generation of the acoustic maps. In this case, any array geometry of any given size and with any orientation can be used for the acoustic beamforming. In consequence, the microphone array used in the CAB methodology can be placed at any location in order to achieve optimal performance for the noise source localization.

An examination of Figures 17 and 18 suggests that the identified noise source locations are different at different frequencies. This implies that the physical mechanisms responsible for the noise generation at these frequencies are different. For frequencies between 200 and 400 Hz, the identified noise source is located in the central area of the rotor plane. The noise at this location might be due to the wind turbine nacelle. As the frequency increases, the location of the identified noise source shifts downwards from the nacelle towards the outer portion of the rotor plane. The source of this noise (which is generated in the frequency range between 400 and 630 Hz) probably arises from the blade tower interaction. This inboard noise caused by the turbine nacelle and blade tower interaction has also been observed and reported in the literature [34, 35].

However, at frequencies below 200 Hz, the noise source location is difficult to identify because this source appears to cover a relatively large area in the acoustic maps. It is anticipated that the use of more advanced acoustic beamforming techniques can potentially be used to improve the spatial resolution and, hence, to better localize the noise sound source in the acoustic maps for frequencies less than 200 Hz. Two examples of super-resolution techniques for acoustic beamforming are the nonnegative least squares and the deconvolution approaches for noise source identification. It has been reported that these super-resolution techniques can improve the spatial resolution of the acoustic maps by a factor typically between three and ten [36].

To better localize the noise sources in the acoustic maps for frequencies above 800 Hz for this small wind turbine, a finer mesh and a smaller time step will need to be used in the CFD simulations. If this is done, it is anticipated that the high-frequency noise source information (for frequencies greater than about 800 Hz) can be encapsulated in the flow field calculations. This information can subsequently be passed onto the aeroacoustic calculations which can then be used in the generation of acoustic maps that will provide better localization of noise sources from the wind turbine that are associated with frequencies greater than about 800 Hz.

## 5. Conclusion

We have proposed the CAB methodology for identification of noise sources generated by a small wind turbine. This predictive method was validated using the NACA 0012 airfoil trailing edge noise case. The predicted acoustic maps obtained using the methodology were in excellent agreement with the corresponding observed acoustic maps obtained from wind-tunnel experiments. We found that the spatial resolution of the CAB methodology for the acoustic maps increases with increasing frequency. Furthermore, we found that an Archimedean spiral array has a better spatial resolution than a star array for all frequencies of interest. Finally, an Archimedean spiral microphone array exhibits better SNR at frequencies below 1000 Hz, but poorer SNR at frequencies above 1000 Hz when compared to the performance of a star microphone array.

The good agreement with the experimental data for the NACA 0012 airfoil case provides the confidence to apply the CAB methodology on a commercial small wind turbine (WINPhase 10 wind turbine). Despite the coarse grid and large time step used in the CFD simulations, the simulated aerodynamic results (wind turbine power output) and aeroacoustic results (A-weighted SPL spectra) were in good agreement with some field measurements for this wind turbine. The simulated acoustic maps revealed that the blade tower interaction and the wind turbine nacelle were two possible noise generation mechanisms in the range of frequencies between 200 and 630 Hz for this small wind turbine.

The agreement between the numerical results obtained using the CAB methodology and the corresponding experimental data for both the NACA 0012 airfoil and the WINPhase 10 wind turbine suggests that the methodology proposed herein can be used to obtain deeper insights for the noise generation issue for other types of wind turbines and turbomachinery. This is especially true for applications where it would be difficult and expensive to conduct a comprehensive set of acoustic beamforming measurements. The CAB methodology can also be applied in cases that require large-sized microphone arrays and/or large numbers of microphones. In particular, it is anticipated that the CAB methodology will be less expensive to apply in these cases owing to the increasing availability of cheap high-performance computing. In addition, the CAB methodology can also be applied as a virtual proving ground for optimization of microphone array geometries and acoustic beamforming algorithms for noise source identification that can take the user through the complete development cycle from design to evaluation. Finally, the use of the CAB methodology provides not only the acoustic maps for the noise source identification, but also the associated flow field which embodies the sound source information. This additional flow field information, which cannot be provided by the traditional acoustic beamforming experiments, can help researchers to gain deeper physical insights into the causes of the noise generated by turbomachinery (e.g., wind turbines, airfoils, etc.).

## Figures and Tables

**Table 1:** Lift and drag coefficients comparison for the NACA 0012 airfoil at zero degree angle-of-attack.

	Experiment [23, 24]	Simulation (time-averaged)
*c*_*L*_	0	0.0105
*c*_*D*_	(0.0062, 0.0082)	0.0065

## Nomenclature

*b*(*t*): Time-domain beamforming at time *t*
*b*(*ϑ*_*i*_): Time-domain beamforming at grid point *i* at the source plane
*C*: Airfoil chord length
*c*: Speed of sound
*c*_*L*_: Time-averaged airfoil lift coefficient
*c*_*D*_: Time-averaged airfoil drag coefficient
*D*: Wind turbine rotor diameter
*H*: Wind turbine hub height
*L*: Measurement distance for the acoustic beamforming
*L*_*i*_: Component of vector defined in (4)
*L*_*M*_: *L*_*i*_ *M*_*i*_
*L*_*r*_: *L*_*i*_ *r*_*i*_
*M*: Magnitude of local Mach number vector for the source
*M*_*i*_: Component of local Mach number vector for the source defined in Equation (4)
*M*_*r*_: Mach number for the source in the direction of the radiation, *M*_*i*_*r*_*i*_
Ma: Mach number
MI: Total number of microphones
*n*_*i*_: Component of unit outward normal vector to surface
Δ*P*_*ij*_: Local force intensity
*p*′: Gauge pressure
*p*_*m*_: Pressure signal for microphone *m*
*R*_0_: Distance between the microphone array center and the wind turbine
*R*_BF_: Spatial resolution for the acoustic beamforming
Re: Reynolds number
*r*: Distance between the observer and the source
*r*_*i*_: Component of unit vector in the direction of the radiation propagation
*s*: Distance between the assumed source and the microphone array center
*s*_*m*_: Distance between the assumed source and microphone *m*
*t*: Observation time
*t* ^*∗*^: Nondimensional time step
*U*_*i*_: Components of vector defined in (4)
*U*_*n*_: *U*_*i*_ *n*_*i*_
*U*_ref_: Reference velocity at the computational domain inlet
*u*_*i*_: Component of local fluid velocity
*u*_*n*_: Fluid velocity in the normal direction to the body
*u*_*τ*_: Friction velocity
*ν*: Kinematic viscosity of the fluid
*v*_*i*_: Component of local velocity on the body
*v*_*n*_: Local velocity on the body in the direction normal to the body surface
*v*_ref_: Reference velocity for power-law inlet boundary condition prescription
*w*_*m*_: Weighting coefficient for microphone *m*
**x**: Observation position vector
*y*: Distance normal to the wall
*y* ^+^: Dimensionless wall normal distance (≡*u*_*τ*_*y*/*ν*)
**y**: Source position vector
〈 〉: Time average
^—^: Generalized function
$\dot{\;\;}$: Source time differentiation.

### Greek Letters

*ϑ*_*i*_: Grid point *i* on the source plane
*λ*: Wavelength
*ρ*: Density of fluid
*ρ*′: Density perturbation, *ρ* − *ρ*_0_
*τ*: Source time
Δ_*im*_: Propagation time from source plane grid point *i* to microphone *m*
Δ_*m*_: Time delay associated with microphone *m* for spherical wave incidence
Π: Microphone array diameter.

#### Subscripts

0: Fluid variable in quiescent medium
*L*: Loading noise component
*T*: Thickness noise component.

## References

[1] Vanhaeverbeke S. (2007). *Simulation of aeroacoustic emission for small wind turbines [Project thesis]*.

[2] Cho T., Kim C., Lee D. (2010). Acoustic measurement for 12% scaled model of NREL Phase VI wind turbine by using beamforming. *Current Applied Physics*.

[3] Bale A., Johnson D. A. (2013). The application of a MEMS microphone phased array to aeroacoustics of small wind turbines. *Wind Engineering*.

[4] Bale A. E. (2011). *The application of MEMS microphone arrays to aeroacoustic measurements [M.S. thesis]*.

[5] Li C. S. (2014). *Computational acoustic beamforming of noise source on wind turbine airfoil [M.S. thesis]*.

[6] Hutcheson F., Brooks T. (2006). Effects of angle of attack and velocity on trailing edge noise determined using microphone array measurements. *International Journal of Aeroacoustics*.

[7] Devenport W., Burdisso R. A., Camargo H. (2010). Aeroacoustic testing of wind turbine airfoils.

[8] Brooks T. F., Humphreys W. M. Effect of directional array size on the measurement of airframe noise components.

[9] Sarradj E., Schulze C., Zeibig A. Aspects of source separation in beamforming.

[10] Wang M., Freund J. B., Lele S. K. (2006). Computational prediction of flow-generated sound. *Annual Review of Fluid Mechanics*.

[11] Garnier E., Adams N., Sagaut P. (2009). *Large Eddy Simulation for Compressible Flows*.

[12] Smagorinsky J. (1963). General circulation experiments with the primitive equations: part I, the basic experiment. *Monthly Weather Review*.

[13] Van Driest E. R. (1956). On turbulent flow near a wall. *Journal of the Aeronautical Sciences*.

[14] Spalart P. R., Deck S., Shur M. L., Squires K. D., Strelets M. K., Travin A. (2006). A new version of detached-eddy simulation, resistant to ambiguous grid densities. *Theoretical and Computational Fluid Dynamics*.

[15] Spalart P., Allmaras S. (1992). A one-equation turbulence model for aerodynamic flows.

[16] Shur M. L., Strelets M. K., Travin A. K., Spalart P. R. (2000). Turbulence modeling in rotating and curved channels: assessing the Spalart-Shur correction. *AIAA Journal*.

[17] Rumsey C. L., Allison D. O., Biedron R. T. (2001). CFD sensitivity analysis of a modern civil transport near buffet-onset conditions.

[18] Ffowcs Williams J. E., Hawkings D. L. (1969). Sound generation by turbulence and surfaces in arbitrary motion. *Philosophical Transactions of the Royal Society of London: Series A Mathematical and Physical Sciences*.

[19] Christensen J. J., Hald J. (2004). Technical review—beamforming.

[20] Kern M., Opfer H. Enhancement of the dynamic range in acoustic photos by modified time domain beamforming.

[21] Dougherty R. P. Advanced time-domain beamforming techniques.

[22] Migliore P., Oerlemans S. Wind tunnel aeroacoustic tests of six airfoils for use on small wind turbines.

[23] Gregory N., O'Reilly C. L. (1970). Low-speed aerodynamic characteristics of NACA 0012 aerofoil sections, including the effects of upper-surface roughness simulation hoar frost.

[24] Ladson C. L. (1988). Effects of independent variation of Mach and Reynolds Numbers on the low-speed aerodynamic characteristics of the NACA 0012 airfoil section. *Technical Report NASA*.

[25] Rossing T. (2015). *Springer Handbook of Acoustics*.

[26] Patel K. S., Patel S. B., Patel U. B., Ahuja P. A. (2014). CFD analysis of an aerofoil. *International Journal of Engineering Research*.

[27] Ma P., Lien F., Yee E. (2017). Coarse-resolution numerical prediction of small wind turbine noise with validation against field measurements. *Renewable Energy*.

[28] Liu B. (2012). *Acoustic Measurement of WINForce 10 kW Wind Turbine*.

[29] Tu J., Yeoh G. H., Liu C. (2012). *Computational Fluid Dynamics: A Practical Approach*.

[30] International Electrotechnical Commission (2011). Wind turbine generator systems—part 11: acoustic noise measurement techniques. *IEC*.

[31] American Wind Energy Association (2009). Acoustic sound testing. *AWEA Small Wind Turbine Performance and Safety Standard*.

[32] Waller G. C. Prediction of flap-edge noise using STAR-CD.

[33] Ramachandran R. C., Patel H., Raman G., Dougherty R. P. Localization of wind turbine noise sources using a compact microphone array with advanced beamforming algorithms.

[34] Oerlemans S., Schepers J. G. (2009). Prediction of wind turbine noise and validation against experiment.

[35] Simley E. J. (2010). *Development of an acoustic array for wind turbine aeroacoustic noise analysis [M.S. thesis]*.

[36] Ginn K. B., Haddad K. Noise source identification techniques: simple to advanced applications.

